# The origin of autoimmune diseases: is there a role for ancestral HLA-II haplotypes in immune hyperactivity

**DOI:** 10.3389/fimmu.2025.1710571

**Published:** 2025-12-04

**Authors:** Manuel Ruiz-Pablos, Bruno Paiva, Aintzane Zabaleta

**Affiliations:** 1 Faculty of Biological Sciences, Universidad Complutense de Madrid, Madrid, Spain; 2 Clinica Universidad de Navarra, Centro de Investigación Médica Aplicada (CIMA), IdiSNA, Instituto de Investigación Sanitaria de Navarra, Pamplona, Spain

**Keywords:** autoimmunity, HLA class II, DRB1, DQB1, cytokines, immune tolerance, evolutionary genetics, pathogen selection

## Abstract

The prevalence of autoimmune diseases in contemporary human populations poses a challenge for both medicine and evolutionary biology. This review explores how the ancestral human leukocyte antigen class II (HLA-II) haplotypes DR2-DQ6, DR4-DQ8 and DR3-DQ2 could play a central role in susceptibility to these diseases. We propose that these haplotypes, selected in historical contexts of high infectious pressure, may have been maintained because of their ability to elicit strong T-cell responses against pathogens; however, that antigenic promiscuity may be associated with an increased tendency toward immune hyperreactivity in modern environments. This hyperreactivity, involving proinflammatory cytokines including interferon-gamma (IFN-γ), could contribute to the breakdown of tolerance and the emergence of autoimmunity and related clinical phenomena (e.g., Long COVID, myalgic encephalomyelitis/chronic fatigue syndrome and post-vaccination syndromes), although the evidence for the latter remains limited. Finally, we discuss how chronic infections, immunotherapies, vaccination, obesity and chronic physical stressors may exacerbate this susceptibility and consider the therapeutic implications of integrating HLA-II profiling into clinical practice.

## 1 Introduction

Autoimmune diseases, such as multiple sclerosis, rheumatoid arthritis, celiac disease, systemic lupus erythematosus, type 1 diabetes and Sjögren’s syndrome, represent an evolutionary paradox: they are harmful to the organism, yet they are linked to genes that have persisted throughout human evolution (1–3). The principal system involved in this paradox is HLA-II, which plays a central role in antigen presentation and CD4+ T-cell activation (2).

Autoreactive lymphocytes can arise stochastically through T-cell receptor (TCR) and B-cell receptor (BCR) gene rearrangements (4, 5), as well as by somatic hypermutation in B cells (6, 7). These processes occur physiologically during thymic selection and within germinal centers (7–9), and may be further amplified in ectopic lymphoid structures under conditions of chronic inflammation (10, 11). In this context, both self-antigens and neoantigens, peptides generated by somatic mutations or post-translational modifications, can be presented by HLA-II molecules, potentially triggering the activation of autoreactive clones in genetically susceptible individuals (12–14). Whether such clones remain quiescent or progress to pathogenicity depends largely on the peptide-binding repertoire of HLA class II molecules (12, 13, 15, 16).

In this context, the ancestral HLA-II haplotypes DR2-DQ6, DR4-DQ8 and DR3-DQ2 (corresponding, respectively, to HLA-DRB1*15:01-DQA1*01:02-DQB1*06:02, HLA-DRB1*04:01-DQA1*03:01-DQB1*03:02 and HLA-DRB1*03:01-DQA1*05:01-DQB1*02:01) stand out for their evolutionary capacity to confront lethal pathogens, but also for their association with autoimmune diseases (1, 2, 17–20).

This review examines how these ancestral haplotypes have contributed to human survival against infections and how their antigenic promiscuity translates into an increased risk of autoimmune disease. We also explore modern triggers, such as chronic infections, obesity, physical trauma, immunotherapies and vaccines, that exacerbate this risk in individuals carrying these haplotypes.

**Figure 1:**
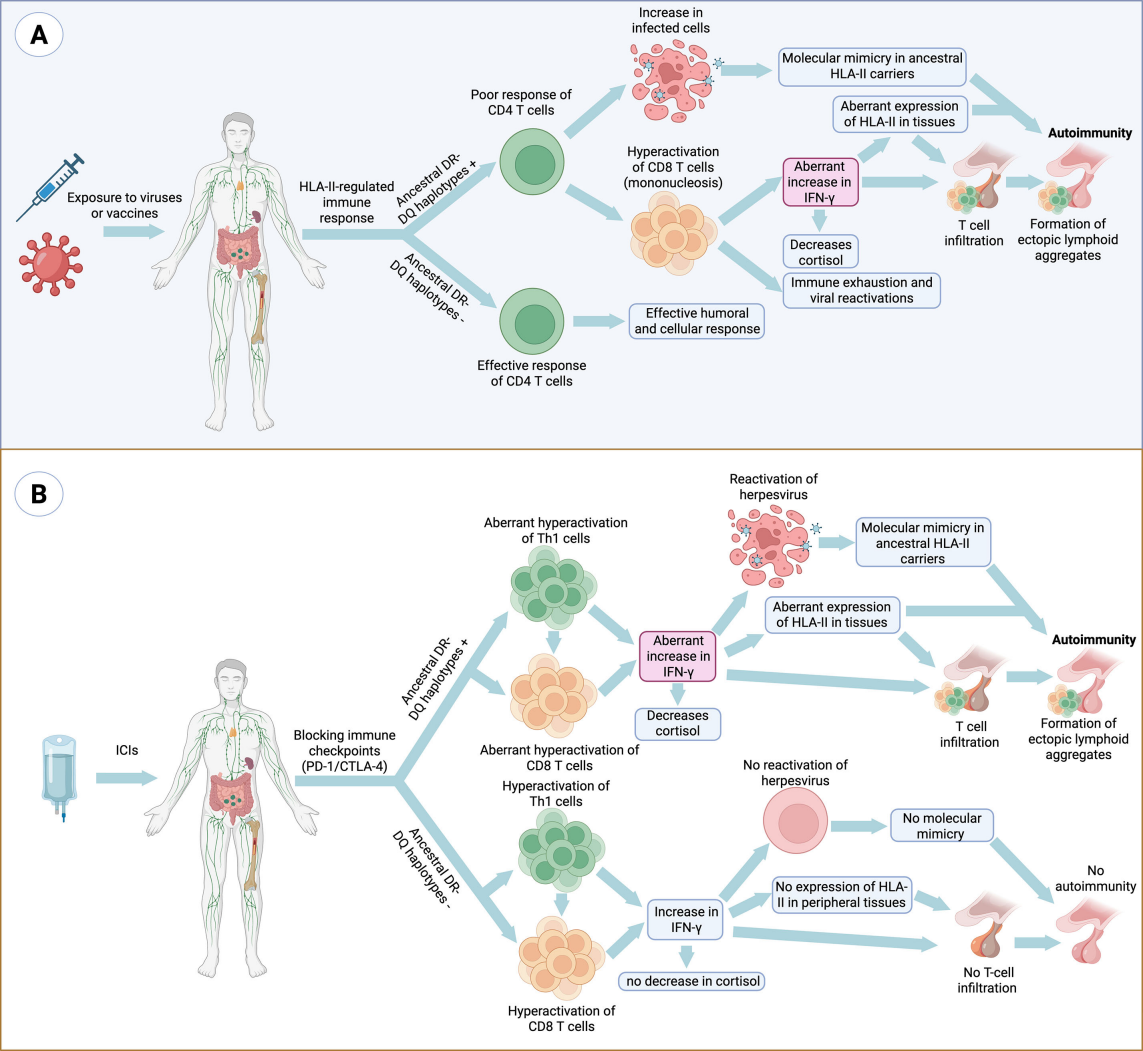
Mechanisms of autoimmunity mediated by ancestral HLA class II haplotypes in response to viral infections, vaccination and immune checkpoint inhibition. **(A)** Certain vaccine-derived antigens or pathogens with antigen persistence and immune-evasion strategies, such as Epstein–Barr virus, may display in carriers of ancestral HLA class II haplotypes (DR2–DQ6, DR3–DQ2 and DR4–DQ8) a limited CD4⁺ T-cell recognition of specific epitopes. This limitation favors antigen persistence and the compensatory expansion of CD8⁺ T cells. Chronic stimulation and sustained interferon-γ production by CD8⁺ T cells and NK cells promote ectopic HLA class II expression, molecular mimicry, the formation of lymphoid aggregates and an increase in antigen presentation to CD4⁺ T cells. Moreover, ancestral HLA class II haplotypes exhibit greater peptide-binding promiscuity, including binding of self-peptides through molecular mimicry, and generate stronger Th1 responses (IFN-γ) compared with other haplotypes, which is associated with reduced cortisol levels and the establishment of a sustained pro-inflammatory microenvironment. The combination of chronic immune hyperreactivity and persistent antigenic stimulation progressively leads to immune exhaustion and loss of tolerance, thereby promoting autoimmunity. **(B)** A parallel mechanism occurs following immune checkpoint blockade (ICIs, anti-PD-1/anti-CTLA-4), where T-cell disinhibition exacerbates hyperactivation in carriers of ancestral HLA haplotypes. This favors viral reactivation, molecular mimicry and aberrant HLA-II expression in peripheral tissues, thereby amplifying T-cell infiltration, ectopic lymphoid aggregate formation and the risk of autoimmunity.

We propose an integrative model linking HLA class II haplotypes, antigen persistence, and the breakdown of immune tolerance (**Figure 1**).

## 2 Search strategy and selection criteria

This manuscript is a narrative review based on structured literature searches performed principally in PubMed, supplemented by searches in Scopus, Google Scholar, and preprint servers (medRxiv/bioRxiv) up to 30 September 2025. Search strategies combined controlled vocabulary (MeSH) and free-text terms using Boolean operators. Key terms and their combinations included concepts covering HLA class II haplotypes (e.g., “HLA class II”, “HLA-DRB1”, “DQB1”, “DR2-DQ6”, “DR3-DQ2”, “DR4-DQ8”), autoimmunity (e.g., “autoimmune disease”, “multiple sclerosis”, “type 1 diabetes”, “rheumatoid arthritis”, “systemic lupus erythematosus”, “celiac disease”), infectious triggers and chronic infections (e.g., “EBV”, “CMV”, “Mycobacterium tuberculosis”, “chronic infection”), immunotherapies and vaccines (e.g., “checkpoint inhibitor”, “immune-related adverse events”, “vaccine”, “adjuvant”), and related mechanistic topics (e.g., “immune exhaustion”, “IFN-gamma”, “HLA phylogeny”, “antigenic promiscuity”, “molecular mimicry”), among others.

Inclusion criteria comprised original research articles, mechanistic animal studies (including humanized HLA transgenic models), systematic reviews and meta-analyses, and relevant clinical reports that addressed the immunogenetic or evolutionary role of HLA-II haplotypes in infection or autoimmunity. A single preprint from medRxiv (21) met the inclusion criteria and is identified as a preprint in the reference list. Exclusion criteria included articles not available in English and publications without primary immunogenetic or mechanistic relevance to the topics addressed.

Titles and abstracts were screened by the authors, and full texts of potentially relevant articles were reviewed. Additional references were identified by manual reference-list screening of key publications. The aim was not to conduct a systematic review or meta-analysis but to produce an integrative, up-to-date narrative synthesis of available evidence linking ancestral HLA-II haplotypes to immune hyperreactivity and autoimmunity.

## 3 Evolutionary context of HLA haplotypes

Human evolution has been shaped by multiple environmental, pathogenic and social factors that favored the selection of genetic variants that maximize survival against diverse threats, particularly infectious diseases (1, 20, 22). Among these variants, HLA haplotypes occupy a central position because they are involved in antigen presentation to T cells and thereby mediate the immune response to pathogens (1, 20). The haplotypes DR2-DQ6, DR4-DQ8 and DR3-DQ2 are among the most prevalent in Caucasian human populations (19, 23–25) and have a complex evolutionary history, linked both to resistance to lethal infections in the past and to susceptibility to autoimmune diseases in the present (1, 20, 22). This relationship poses an evolutionary paradox: haplotypes that in a primitive environment conferred a survival advantage against acute extracellular infections may, in the modern context of increased life expectancy, persistent latent infections, widespread vaccination, immunotherapies, and proinflammatory conditions such as obesity, instead predispose to harmful autoimmune responses.

These three ancestral haplotypes account for approximately 90% of autoimmune diseases (20), including type 1 diabetes, as recently confirmed by a multi-ancestry genome-wide association study (GWAS) (26). Given that thousands of HLA-II haplotypes exist in the human population, it is intriguing to understand why these three haplotypes are particularly associated with autoimmunity (1, 20). If these genes were broadly deleterious, natural selection would likely have eliminated them over time (20). Instead, their high allele frequencies in autoimmunity suggest that they were advantageous in historical contexts, being essential for species survival under those conditions (20). It is hypothesized that, over millennia, these genes accumulated variants enabling them to present a wider diversity of pathogen-derived peptides, thereby activating larger numbers of CD4+ T cells to defend the host against infection (20). However, we propose that the alternative hypothesis advanced by Mangalam et al. is more compelling: because these haplotypes are ancestral, they may have been inherently more promiscuous in antigen recognition from their origins, which allowed them to persist through evolutionary bottlenecks (1). This promiscuous antigen recognition is illustrated in Mangalam et al., which show that epitopes presented by certain HLA-DR alleles (for example, HLA-DRB1*15:01) have a broader binding capacity not only to autoantigenic peptides but also to infectious agents, such as hepatitis viruses (1, 20). Similar findings have been reported for the alleles HLA-DRB1*03:01 and HLA-DRB1*04:01 (1, 20). Such promiscuity in the recognition of pathogen-derived peptides would have conferred a crucial adaptive advantage for species protection throughout history.

## 4 HLA-DRB1 lineages and their phylogenetic history

In humans there are 13 allelic lineages of HLA-DRB1 (22). According to the phylogenetic relationships among DRB genes in primates (hominoids, New World monkeys and Old-World monkeys) described by Bontrop et al., the HLA-DRB1*04, *03 and *02 lineages are the oldest, with HLA-DRB1*04 being the most ancestral lineage (27). Yasukochi and Satta identified two main groups of HLA-DRB1 alleles, designated Group A and Group B, based on a phylogenetic analysis (22). The alleles HLA-DRB1*04 and HLA-DRB1*15 belong to Group B, which is characterized by trans-species polymorphism (22). These lineages, shared with chimpanzees and other primates (for example Patr-DRB1*02 and Patr-DRB1*07), have a very ancient origin, predating the human–chimpanzee divergence approximately 6–7 million years ago (22). By contrast, HLA-DRB1*03 is associated with Group A, which has a more recent origin (22).

Phylogenetic data indicate that Group B is older, with an estimated time to the most recent common ancestor (TMRCA) of about 41 million years, whereas Group A has a TMRCA of approximately 29 million years (22). This pattern suggests that alleles such as HLA-DRB1*04 and HLA-DRB1*15 have been maintained by balancing selection because of their functional importance in immune responses (22). Although HLA-DRB1*03 emerged more recently, it has also been conserved due to its functional relevance (22). Moreover, the HLA-DRB1*03 lineage has been identified in humans, chimpanzees, bonobos, gorillas and orangutans, reinforcing its antiquity (28). A putative “proto-HLA-DRB1*03” ancestral lineage appears to have diverged in the last ~5 million years, giving rise to human-specific lineages such as HLA-DRB1*08, *11, *13 and *14 (28).

Taken together, these phylogenetic trees are consistent with the antiquity of these alleles and with the possibility that their long-term conservation reflects functional importance for immune responses. The HLA-DRB1*04 (DR4) and HLA-DRB1*15 (DR2) lineages are among the most ancient, followed by HLA-DRB1*03 (DR3), which is somewhat more recent. The persistence of these lineages over millions of years underscores their effectiveness against acute infections and their capacity to recognize a broader repertoire of pathogen antigens.

### 4.1 Consolidation of ancestral HLA haplotypes: genetic bottlenecks and positive selection

The study of the evolution of HLA haplotypes in *Homo sapiens* and their relationship with adaptation to pathogens over time is essential to understand how natural selection has shaped human genetic diversity (1, 20, 22). Diversity among HLA molecules has been maintained through long-term host–pathogen coevolution, with HLA loci being the most polymorphic in the human genome (1, 20, 22). This diversity is reflected in the persistence of allelic lineages and the sharing of trans-species polymorphisms between closely related species, such as chimpanzees and humans (22). Modern humans, after their exodus from Africa, encountered novel pathogen repertoires outside their continent of origin, which may have favored local positive selection of certain HLA alleles, as observed in patterns of ancestral haplotype frequencies across different populations (1, 20, 22).

During the evolution of *Homo sapiens*, ancestral HLA haplotypes became established primarily due to their ability to present key pathogen-derived antigens that affected early human populations (1, 20, 22). This consolidation, influenced by genetic bottlenecks such as migrations and glaciations, resulted in the selection of genetic variants that improved survival against lethal infections (1, 20). However, it also contributed to a reduction in genetic diversity that has persisted to the present.

Migration out of Africa (~50,000–55,000 years ago):

As humans migrated into Asia, Europe, and other continents, they encountered new pathogens and environmental conditions (1, 29). In this context, positive selection favored HLA alleles that maximized immune responses against these local pathogens (1).

Historical pandemics:

At various times in human history, pandemics have exerted profound impacts on the selection of genetic variants (30). The Black Death (1346–1353) imposed strong selective pressure on the human immune system, altering the frequency of several class II HLA alleles, some of which increased in frequency such as HLA-DRB1*13, while others decreased following the pandemic (31). However, the HLA-DRB1*15:01 and HLA-DRB1*03:01 haplotypes not only remained stable in frequency before and after the outbreak, but were also among the most frequent alleles in the population at both times, which could indicate that they already provided a sufficient level of protection against *Yersinia pestis*, thereby avoiding additional selective pressure on them (31). This stability in their prevalence over time supports the hypothesis that these ancestral haplotypes have been effective in immune responses against multiple pathogens.

Similarly, influenza virus has been a key factor in the evolution of the HLA repertoire (32). The allele HLA-DRB1*04:01 has been observed to confer greater resistance to influenza (H1N1) by eliciting robust immune responses through more effective T cell activation and increased interferon-gamma (IFN-γ) production (32). In animal model studies, individuals carrying HLA-DRB1*04:01 showed faster recovery and greater cross-protection against different influenza strains compared with the allele HLA-DRB1*04:02 (32). This immunological advantage could have favored its selection over time, despite its association with autoimmune diseases such as rheumatoid arthritis (33). Similarly, during the 1918 Spanish influenza pandemic, ancestral haplotypes were observed to influence the severity of respiratory infections (34, 35). In this context, HLA-DRB1*04:01 and HLA-DRB1*03:01, by conferring greater cross-immunity with different influenza strains, could have provided an additional immunological advantage against the Spanish influenza virus, favoring their selection in subsequent generations (35). A more recent study reinforces this hypothesis by demonstrating that immunization with conserved CD4+ T-cell epitopes between the 2009 pandemic H1N1 and prior seasonal strains reduced viral load in HLA-DR3 transgenic mouse models (35). This suggests that cell-mediated immunity driven by these alleles can confer partial protection against infection and modulate disease severity, even in the absence of cross-reactive antibodies.

However, not all historical cases show a protective effect for these alleles. In the case of medieval leprosy, an ancient DNA study of skeletal remains from infected individuals at the St. Jørgen leprosarium (Odense, Denmark) showed that the allele HLA-DRB1*15:01, rather than conferring protection, was significantly associated with increased susceptibility to lepromatous leprosy, similar to observations in modern populations in India, China, and Brazil (36). Nonetheless, this allele persisted in the population, suggesting that it may have conferred immunological advantages in other infectious contexts.

### 4.2 Genetic variability and adaptation of the HLA class II system

The HLA class II system, located on chromosome 6, is notable for its high genetic variability, which reflects positive selection exerted by pathogen exposure throughout human evolution (1, 20, 22). In African populations, where Homo sapiens originated, there is greater diversity of HLA class II alleles (1, 22). As humans migrated out of Africa, they faced novel pathogens and population bottlenecks that favored the selection of specific haplotypes such as DR2-DQ6, DR4-DQ8, and DR3-DQ2, which were particularly effective at presenting pathogen antigens and eliciting robust immune responses (1, 2, 17–20, 22). These haplotypes facilitated efficient activation of CD4+ T lymphocytes and the secretion of proinflammatory cytokines such as IFN-γ and IL-17, which are essential for survival during viral and bacterial epidemics (1, 20, 32, 37, 38). Moreover, coexpression of HLA class II molecules allows individuals who are homozygous or heterozygous for ancestral haplotypes to experience a synergistic effect, increasing T cell activation and the secretion of inflammatory cytokines (1).

However, this capacity to recognize a broad diversity of antigens, although advantageous from an evolutionary standpoint, carries a dual impact. In the modern environment, characterized by reduced mortality from acute infections due to medical advances, but increased exposure to chronic immunological stimuli (persistent infections, vaccinations, immunotherapies), this capacity may predispose carriers of these haplotypes to an exaggerated inflammatory response and the development of autoimmune diseases (20). Indeed, these HLA class II variants could contribute to dysregulated immune activation in the presence of a chronic triggering stimulus (e.g., persistent infections), creating an immunological imbalance that favors autoimmunity in genetically susceptible individuals. This persistence is consistent with host–pathogen co-evolution: many viruses have selected immune-evasion and persistence strategies (latency or long-term infections) that favor coexistence with the host rather than lethality, producing continuous antigenic exposure that can be deleterious when it coincides with promiscuous HLA-class-II haplotypes (12, 39–41) rather than lethality, producing continuous antigenic exposure that can be deleterious when it coincides with promiscuous HLA-class-II haplotypes (12, 39–41).

### 4.3 The survival paradox: from protection to autoimmunity

**Figure 2:**
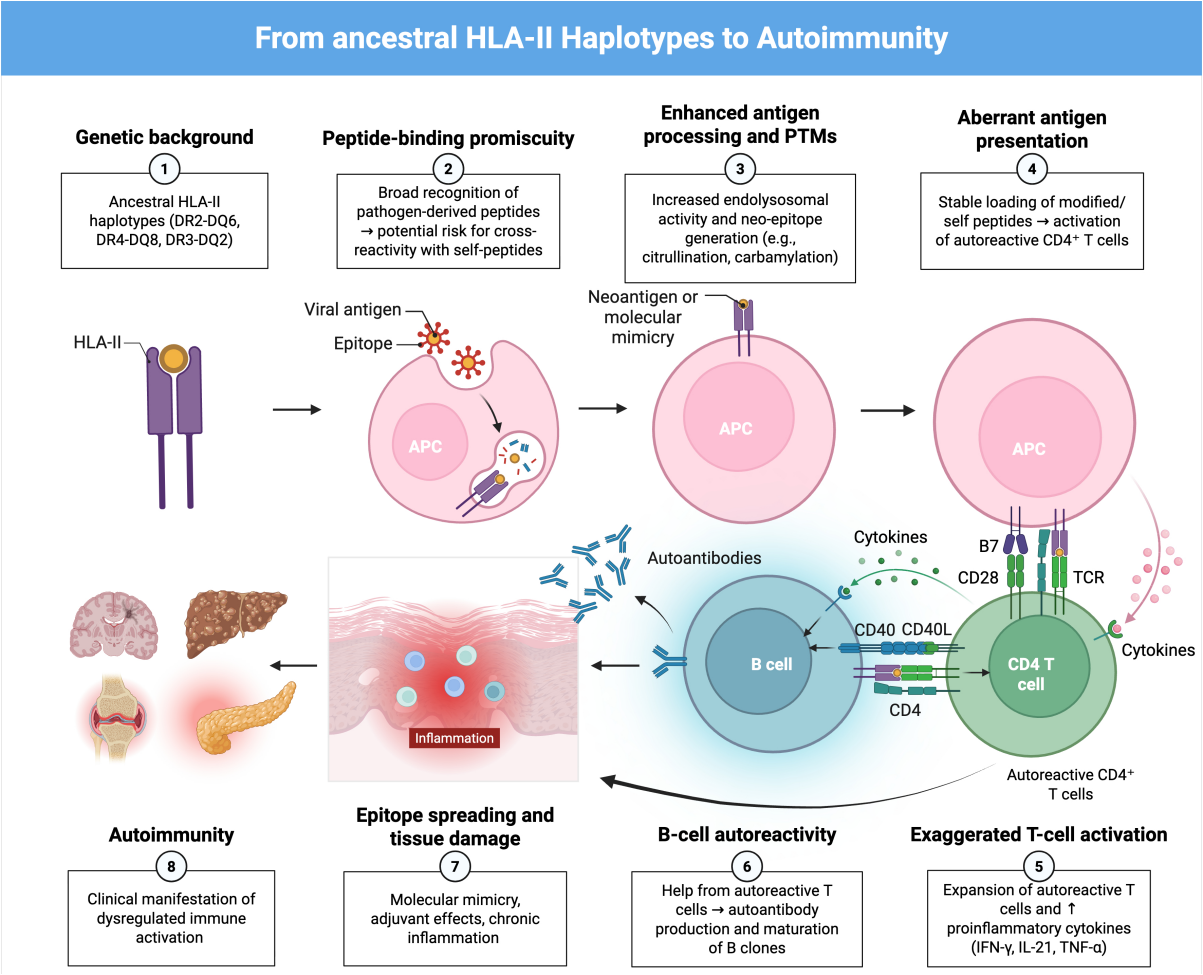
Proposed pathway of autoimmunity driven by ancestral HLA-II haplotypes.Ancestral HLA-II haplotypes (such as DR2-DQ6, DR4-DQ8, and DR3-DQ2) exhibit high peptide-binding promiscuity, facilitating the presentation of a wide variety of pathogen-derived peptides. Inflammation induced by these infections, particularly through IFN-γ, together with the generation of neoantigens via post-translational modifications, stimulates MHC-II expression even in non-professional antigen-presenting cells and increases the presentation of self-peptides, which can lead to the activation of autoreactive CD4^-^ T cells via TCR-MHC-II recognition and B7-CD28 co-stimulation. Activated T cells proliferate and secrete proinflammatory cytokines (IFN-γ, IL-21, TNF-α), amplifying inflammation and promoting B-cell autoreactivity. These B cells present the same antigen on MHC-II and receive help from T cells through CD40-CD40L interactions, driving their differentiation into memory and plasma cells that produce autoantibodies. The combination of epitope spreading, molecular mimicry, and adjuvant-induced activation leads to chronic inflammation, tissue damage, and the clinical manifestation of autoimmune diseases.

The survival paradox lies in the fact that the same haplotypes that conferred an evolutionary advantage against lethal infections in the past can now generate harmful autoimmune responses (1, 19, 20, 22). Haplotypes DR2-DQ6, DR4-DQ8 and DR3-DQ2 were favored for their ability to elicit effective immune responses against deadly infections, but in a modern environment, where lethal infections are less common and chronic stimuli such as latent infections or systemic inflammation predominate, these haplotypes may contribute to increased risk of autoimmunity (1, 19, 20, 22). Crucially, the emergence and persistence of pathogenic autoreactive clones depends not only on HLA-II–restricted peptide presentation but also on the host T- and B-cell receptor repertoire: although TCR and BCR sequences are generated stochastically by V(D)J recombination (and by somatic hypermutation in B cells), HLA-dependent selection processes in the thymus and in germinal centers strongly shape which clones are deleted, tolerated or expanded, such that HLA alleles indirectly influence which autoreactive clones survive and potentially become pathogenic (42–46). In other words, the persistence of these haplotypes suggests an adaptive balance in which the immune response that provides protection against acute infections can become hyperreactive and increase autoimmune risk in the presence of chronic infections (**Figure 2**).

Molecular-level mechanisms can reconcile how an evolutionarily advantageous HLA-II repertoire becomes pathogenic in modern contexts (13). Allele-specific peptide-binding preferences (determined by the composition of pockets within the HLA-DR/DQ binding groove) result in selective presentation of particular self-peptides or post-translationally modified peptides (e.g. citrullinated or deamidated peptides) that otherwise escape thymic deletion (47). For example, HLA-DQ2/DQ8 preferentially present deamidated gliadin peptides in coeliac disease (48), HLA-DRB1 shared-epitope alleles bind citrullinated vimentin/filaggrin peptides in rheumatoid arthritis (49), and HLA-DQB1*03:02 (DQ8) presents insulin B-chain peptides implicated in type 1 diabetes (50). Concurrently, inflammatory cytokines such as IFN-γ activate the JAK–STAT1–CIITA pathway, driving ectopic upregulation of HLA-II on non-professional antigen presenting cells and facilitating local presentation of these auto-epitopes to autoreactive CD4+ T cells (51). Together, allele-dependent peptide selection and inflammation-driven HLA-II expression provide a molecular route from pathogen-focused immunity to loss of self-tolerance (13).

## 5 Ancestral HLA class II haplotypes and resistance to acute infections

Alleles HLA-DRB1*15, HLA-DRB1*04 and HLA-DRB1*03 have been extensively studied for their capacity to induce intense immune responses (19, 20, 52, 53). These alleles have shown efficacy in clearing rapidly replicating pathogens due to their ability to induce a strong T helper 1 (Th1)-type response mediated by IFN-γ and TNF-α (19, 20, 52).

In dengue virus infection, the ancestral alleles HLA-DRB1*04:01 and HLA-DRB1*15:01 have shown greater resistance to dengue virus infection, recognizing a larger number of epitopes and generating a more efficient IFN-γ response compared with other alleles and closely related allelic variants, such as *04:03 and *15:02 (38). Similarly, infants who inherit HLA-DRB1*15:01 have been observed to have a higher probability of reverting to HIV (human immunodeficiency virus)–seronegative status despite being born with maternal anti-HIV antibodies (54). This phenomenon could be explained by a more effective immune response provided by this allele, possibly via increased IFN-γ production, which would contribute to improved immune control during early exposure. Likewise, in visceral leishmaniasis, the presence of HLA-DRB1*15:01 favors higher IFN-γ/IL-10 ratios, promoting a robust protective Th1 response (37, 55).

Furthermore, the DR2-DQ6 haplotype produces high levels of IFN-γ and is associated with an efficient response against intracellular pathogens such as *Mycobacterium tuberculosis*, a feature particularly critical in regions where tuberculosis was endemic (1). A meta-analysis of HLA class II variability and hepatitis B infection indicated that the HLA-DR4 allele is significantly associated with the ability to clear hepatitis B virus (HBV) (56). Similarly, alleles HLA-DRB1*04:01 and HLA-DRB1*15:01 have been linked to greater efficacy in clearing hepatitis C virus (57).

The study by Rodríguez et al. (2008) demonstrated that transgenic mice expressing HLA-DR2 and HLA-DR3 controlled viral encephalomyelitis induced by Theiler’s murine encephalomyelitis virus (TMEV) through increased production of IFN-γ and IL-2 by CD4+ T cells, allowing effective viral clearance without the need for antigen presentation by HLA class I (52)​. This mechanism is consistent with a scenario in which these haplotypes could have been favored by selection for rapid responses to lethal infections, potentially providing a survival advantage under high pathogen pressure (e.g., historical epidemics and pandemics). Moreover, multiple model systems have shown that IFN-γ is essential for control of this virus in the central nervous system (CNS) (58–60), which supports the hypothesis that haplotypes associated with greater IFN-γ production during acute infection may be more effective at eliminating intracellular pathogens.

These examples support the hypothesis that ancestral HLA class II haplotypes may have contributed to resistance against highly lethal infections, potentially favoring survival in populations exposed to such pathogens (61). However, this same intense activation becomes detrimental in the context of chronic intracellular infections, where pathogens (e.g., herpesviruses such as EBV or CMV, and certain intracellular bacteria) have co-evolved with the host to develop sophisticated immune-evasion strategies, including inhibition of antigen presentation, establishment of latency or persistent reservoirs, and induction of IL-10-dominated immunosuppressive milieus (62–64). Such evasion prevents complete clearance and leads to sustained antigenic stimulation and chronic inflammation (65), contributing to the development of autoimmune diseases such as multiple sclerosis (66). This phenomenon explains why ancestral haplotypes were positively selected in epidemic and pandemic contexts, yet today their hyperreactivity may predispose to autoimmune disorders and poorer regulation of chronic infections.

## 6 Ancestral HLA class II haplotypes and immune hyperreactivity in chronic infections

Ancestral HLA class II haplotypes, such as DR2-DQ6, DR3-DQ2 and DR4-DQ8, have evolved under infectious pressure, which has favored a hyperactive immune response (1, 19, 20, 22). Although this reactivity provides an advantage in detecting intracellular pathogens, its persistence does not always guarantee elimination of the microorganism and, in many cases, leads to chronic inflammation, tissue damage and an increased risk of autoimmune disease (20). The inability of the immune system to eradicate certain pathogens is due to microbial evasion strategies that neutralize or modulate the immune response, allowing their survival within the host despite constant T cell activation (19, 20, 53).

The relationship between HLA haplotypes and susceptibility or resistance to chronic infections, such as hepatitis B and tuberculosis, illustrates this paradox. The DR2-DQ6 haplotype has been observed to exert a protective effect against initial acute hepatitis B infection (67–71). In addition, HLA-DRB1*15:01 is associated with an enhanced humoral response to the hepatitis B surface antigen (HBsAg), promoting more efficient anti-HBsAg antibody production via activation of CD4+ T cells (72). However, this same haplotype has also been associated with an increased risk of chronic infection (73–75). This duality suggests that initial resistance to infection may be linked to an exaggerated immune response that, over time, leads to immune exhaustion and progression to chronic infection.

The hypothesis explaining this paradox is based on the cytokine hyperresponse induced by these ancestral haplotypes during the acute phase of infection. The strong initial immune response facilitates partial viral clearance but fails to eradicate the virus completely because of viral evasion mechanisms. This same hyperimmunity can be exploited by pathogens that have developed evasion strategies, resulting in chronic inflammation and tissue damage. HBV employs strategies such as inhibition of antigen presentation and induction of T cell anergy, which allow it to persist despite a robust initial response (76, 77). This leads to functional exhaustion of T cells and chronic inflammation that favor progression to chronic hepatitis (76).

Similarly, in tuberculosis, certain HLA haplotypes have been linked to protection against initial infection but also to an increased risk of progressive latent tuberculosis (1, 78, 79), certain HLA haplotypes have been linked to protection against initial infection but also to an increased risk of progressive latent tuberculosis (1, 78, 79). This reinforces the idea that the same immunological advantage that protects against initial infection can, under conditions of pathogen persistence due to evasion mechanisms, become a disadvantage by promoting a chronic inflammatory environment that favors the microorganism’s persistence in the host.

These findings underscore the complexity of the interaction between host genetics and the evolution of infectious diseases. The persistence of these haplotypes in the population could be explained by their advantage in protecting against lethal acute infections, despite the cost of increased predisposition to chronicity. This initial evolutionary advantage would have increased the fitness of carrier individuals, allowing them to reach reproductive age and transmit these alleles to their offspring. The fact that these haplotypes have been maintained in the human gene pool suggests that the benefit of effective protection against acute infections outweighed the cost of greater susceptibility to chronic infection and long-term immune exhaustion.

*M. tuberculosis* evades the immune response by blocking phagosome–lysosome fusion within macrophages, thereby permitting intracellular survival (80). Furthermore, within the granuloma it induces production of IL-10 and TGF-β, creating a localized immunosuppressive environment that reduces macrophage and T cell activation and hinders pathogen clearance (80). In this context, the DR2-DQ6 haplotype has been associated with greater susceptibility to pulmonary tuberculosis and a less effective response for controlling the infection (78, 79). The mutation from DQ6.1 to DQ6.2 of the DR2-DQ6 haplotype in European populations increased the production of IFN-γ and IL-17, improving the response to intracellular pathogens (1). However, it also intensified inflammation, which increased susceptibility to autoimmune diseases such as multiple sclerosis (1).

Both *M. tuberculosis* and Epstein-Barr virus (EBV) have been implicated in the induction of autoimmune diseases in individuals carrying the HLA-DRB1*15:01-DQB1*06:02 haplotype (81–86). In the case of EBV, hyperreactivity of HLA class II haplotypes such as HLA-DRB1*15 (12, 13, 15, 16).

In this context, the ancestral HLA-II haplotypes DR2-DQ6,:01-DQB1*06:02 induces intense activation of CD4+ and CD8+ T cells (53, 84). However, EBV evades the immune system by downregulating antigen presentation in infected B cells and promoting an IL-10–mediated immunosuppressive milieu (87). Moreover, this haplotype contributes to poor recognition of EBV-infected cells, further hindering viral clearance (**Figure 1**) (53). As a result, the virus persists in the organism, provoking continuous immune hyperactivation with chronic inflammation that can contribute to the development of autoimmune diseases such as multiple sclerosis (20, 53, 84). In addition, despite CD8+ T cell expansion, their antiviral function is deficient due to inadequate help from HLA- DRB1*15:01-restricted CD4+ T cells, which prevents effective elimination of the virus (53, 84). It should be noted that susceptibility to multiple sclerosis is also observed in other ancestral haplotypes such as DR4-DQ8 and DR3-DQ2 (17).

Similarly, *M. tuberculosis*, by inducing a persistent inflammatory response with elevated IFN-γ and IL-17 production in individuals with the DR2-DQ6 haplotype, can break immune tolerance and contribute to a chronic proinflammatory state that facilitates CNS autoimmunity via molecular mimicry (80, 85, 86, 88). This phenomenon is demonstrated in the experimental autoimmune encephalomyelitis (EAE) model, where *M. tuberculosis* antigens in complete Freund’s adjuvant are used to induce autoimmunity against myelin in susceptible animals, suggesting that prolonged exposure to this pathogen could be an additional environmental factor in the pathogenesis of multiple sclerosis in genetically susceptible individuals (81, 85, 89, 90). This process is related to the cross-restriction by HLA class II and cross-reactivity of CD4+ T cells to hyperstimulatory antigens in infections such as herpes simplex virus type 2, influenza A virus and *M. tuberculosis*, which could favor dysregulated immune responses and contribute to the development of autoimmunity (85, 86, 91).

Cytomegalovirus (CMV) is another pathogen that exploits immune hyperreactivity without being eradicated (92–94). HLA-DRB1*15:01 has been associated with a reduced CD4+ T cell response to the CMV pp65 antigen and with increased susceptibility to CMV infections following hematopoietic stem cell transplantation (95). This suggests that individuals carrying HLA-DRB1*15:01 may have impaired control of CMV, leading to chronic inflammation, accelerated immunosenescence, immune exhaustion and long-term impairment of immune responses (95, 96). The virus elicits a strong immune response, but this hyperreactivity paradoxically leads to immune exhaustion and dysfunction over time (92–94, 97). CMV employs several evasion mechanisms, such as interfering with T cell activation and altering dendritic cell function, which hinder presentation of viral antigens (96, 98, 99). In addition, CMV induces production of IL-10 and TGF-β, creating an immunosuppressive milieu that limits effective T cell activity at sites of infection (96, 99). This chronic activation of the immune system contributes to CD8+ T cell exhaustion, compromises immune surveillance, favors periodic viral reactivation and may promote the development of autoimmunity (92, 96, 97, 100).

In the case of *Mycobacterium leprae*, the pathogen responsible for lepromatous leprosy, its association with HLA-DRB1*15:01 promotes a Th2-type immune response characterized by excessive IL-10 and TGF-β production at foci of infection (36, 101). This blocks macrophage activation and intracellular killing of the pathogen, permitting its proliferation within Schwann cells and establishing a localized state of immunosuppression (101, 102). Despite strong systemic immune hyperreactivity, the infection is perpetuated and results in CD8+ T cell exhaustion and chronic tissue damage, while sustained inflammation favors autoimmunity in susceptible individuals (101).

The alleles HLA-DRB1*15, HLA-DRB1*04 and HLA-DRB1*03 have been associated with increased baseline immune hyperactivity, resulting in constant activation of CD4+ and CD8+ T cells (19, 20, 53). However, pathogen evasion mechanisms, often evolved specifically to blunt host immunity, can impair effective responses regardless of the individual’s HLA-II haplotype, meaning that even hyperreactive haplotypes may fail to clear certain intracellular infections (53, 103). Thus, the common mechanism among these pathogens is the interaction between high HLA-II reactivity and microbial evasion strategies. The capacity of these haplotypes to present a larger number of epitopes favors expansion of CD4^-^ T cells with both increased clonal breadth and heightened effector activity (e.g. IFN-γ); however, this also includes an increased pool of autoreactive T cells that escape thymic deletion (20). This may create a vicious cycle in individuals with hyperreactive HLA-II haplotypes: the immune system detects the pathogen, but, unable to eliminate it completely, immune activation persists. Consequently, chronic activation of CD8+ T cells occurs which, under persistent stimulation, become exhausted and lose functionality (76, 92, 96, 97, 101, 104). In an attempt to compensate for this exhaustion, a shift toward a Th2 response is observed, with increased production of IL-4 and IL-10 and greater B cell activation, which amplifies the humoral response (101, 105). While humoral responses can contribute to pathogen control, effective clearance of many intracellular microbes typically requires Th1-mediated cellular immunity; therefore, a shift toward Th2 may reduce tissue-damaging Th1 activity but can also impair pathogen eradication, favoring persistence (106, 107). This shift not only perpetuates the infection by weakening cellular immunity, but may also promote autoimmunity, since autoantibody production and chronic inflammation maintain dysregulated immune activation. Autoimmunity can arise via failures at multiple checkpoints: defective central tolerance in the thymus, dysregulated germinal-center reactions driving autoreactive B-cell expansion and somatic hypermutation, or ectopic/tertiary lymphoid structures in inflamed tissues that sustain local autoreactive responses (10, 44, 108, 109). Moreover, chronic inflammation associated with T cell exhaustion is also linked to a Th2 cytokine profile within the tumor microenvironment, which could influence cancer progression by promoting an immunosuppressive milieu (101).

Thus, the evolutionary pressure that selected highly reactive and promiscuous haplotypes for defense against acute infections now acts as a predisposing factor for chronic inflammatory and autoimmune diseases in contemporary populations.

In contrast, HLA-II haplotypes with lower functional reactivity may have been favored in certain populations as a strategy of controlled symbiosis, permitting coexistence with chronic pathogens without triggering uncontrolled inflammation. This balance between protection and harm exemplifies how immune system evolution has shaped susceptibility to infectious and autoimmune diseases today.

While evolutionary models provide a coherent framework, quantifying the net fitness trade-offs empirically remains challenging because autoimmune phenotypes typically present post-reproductively and because modern environmental changes (longer lifespan, altered pathogen exposure, demographic shifts) complicate direct measurements of historical selection.

## 7 Persistence of ancestral haplotypes in the present day

The prevalence of these ancestral haplotypes in modern populations reflects not only positive selection exerted by historical infectious pressure but also the persistence of conserved genetic mechanisms (1, 19, 20, 22, 36). Although in a modern context these variants may contribute to autoimmune disease risk, their retention over millennia attests to their importance in protection against lethal pathogens in past environments (1, 19, 20, 22).

For example, DR2-DQ6 has been shown to induce more robust responses to infections such as dengue virus or visceral leishmaniasis, although its prevalence has also been associated with multiple sclerosis (37, 38, 53). DR4-DQ8, while associated with greater resistance to infections such as HBV, has also been linked to autoimmune diseases such as rheumatoid arthritis and type 1 diabetes (33, 56, 110, 111). Similarly, DR3-DQ2 can optimize the immune response to viral infections but is also implicated in autoimmune disorders such as celiac disease and systemic lupus erythematosus (1, 112, 113).

Natural selection therefore favored strong immune responses to lethal infections without accounting for the long-term effects of immune hyperactivation. In other words, natural selection promoted robust immune defenses against acute infections, enabling individuals to reach reproductive age. Autoimmunity, which frequently manifests after reproductive age, likely had a limited impact on evolutionary fitness, facilitating persistence of these alleles. In the modern world, however, factors such as prolonged exposure to latent infections, increased life expectancy and an inflammatory environment exacerbated by pollution and other environmental factors alter this balance, converting ancestral advantages into susceptibilities. From an evolutionary perspective, this phenomenon can be explained as an adaptive trade-off: the immune mechanisms selected ensured immediate survival in a high-infectious-pressure environment, although they resulted in increased autoimmunity later in life, after reproduction had typically occurred.

## 8 Triggering factors of autoimmunity

### 8.1 Chronic infections

This duality raises a key question: how can haplotypes that were evolutionarily selected for their effectiveness in the immune response to acute infections simultaneously cause severe dysfunctions when confronted with pathogens that employ advanced immune-evasion mechanisms, such as latent pathogens, and thereby predispose to the development of autoimmune diseases?

#### 8.1.1 Failure to control latent infections or pathogens with extensive immune-evasion mechanisms

Ancestral HLA haplotypes, such as DR2-DQ6, DR4-DQ8 and DR3-DQ2, represent evolutionary adaptations to acute infections, but in the context of latent infections or pathogens with advanced immune-evasion strategies, such as EBV, their immune responsiveness can be allele-specific attenuated for certain viral epitopes (1, 19, 20, 53). For example, EBV establishes latency in B lymphocytes; in this setting, HLA-DRB1*15:01 (a component of DR2-DQ6) has been associated with attenuated CD4+ T-cell–mediated control of EBV-infected B cells in humanized-mouse models, resulting in higher levels of infected cells and impaired CD4 help to CD8+ responses, which can favor persistence and episodic reactivation of the virus (53). The combination of allele-specific limitations in antiviral CD4 help and sustained antigenic stimulation provides a mechanistic route whereby viral persistence may drive chronic immune activation and increase the risk of autoreactivity.

This dysfunction of the CD4+ T cell response facilitates expansion of virus-transformed cells (latency) and perpetuates low-grade chronic inflammation (53, 84, 100). In carriers of DR2-DQ6 haplotypes, the inability to mount an effective response could create an environment in which the virus alternates more frequently between latency and lytic replication, rather than bei. Unlike other HLA haplotypes that might limit latent viral load and reduce reactivation frequency, DR2-DQ6 favors viral persistence and continuous immune stimulation (53, 114, 115). This phenomenon is not limited to EBV but exemplifies a broader pattern: in carriers of highly reactive haplotypes such as DR2-DQ6, DR4-DQ8 and DR3-DQ2, the mere presence of a latent pathogen may be sufficient to sustain persistent immune hyperactivation due to ongoing antigenic persistence (1, 20, 100, 116, 117).

#### 8.1.2 Compensatory hyperactivation of CD8+ T cells and natural killer cells

In response to deficient CD4+ T cell functionality, the immune system engages alternative pathways to control latent or chronic infection (100, 114, 118, 119). Hyperactivation of CD8+ T cells becomes a critical compensatory response attempting to control the infection (114, 118, 119). CD8+ T cells are responsible for immune surveillance of infected or transformed cells (114). In individuals with HLA haplotypes such as DR2-DQ6, DR4-DQ8, or DR3-DQ2, who have a genetic predisposition to suboptimal CD4+ responses against certain latent pathogens, this dysfunction may force CD8+ T cells to assume a more active role to compensate for the lack of CD4+ control (53, 114). This hypothesis is supported by converging data indicating reduced CD4+ helper capacity in carriers of these haplotypes, higher latent viral loads (as seen in EBV infections), and sustained hyperactivity of the CD8+ compartment under chronic antigenic stimulation (20, 53, 84, 101, 114, 119). This process results in increased production of IFN-γ, a key effector that, while effective against infection, also amplifies inflammation (118).

Concurrently, natural killer (NK) cells, crucial in the early response to viral infections, are activated by cellular stress signals and suboptimal viral antigen presentation (120). IFN-γ production by NK cells further amplifies the inflammatory signal, creating an environment that favors tissue dysfunction (120–122). At this stage, IFN-γ production is therefore dominated by CD8+ T cells and NK cells, while the contribution of CD4+ T cells remains limited, reflecting their secondary role in an imbalanced immune response (123).

#### 8.1.3 Increased compensatory humoral response

As the immune system attempts to compensate for deficient functional CD4+ T cells, particularly Th1 subsets, a shift toward greater activation of CD4+ Th2 cells is observed (101). This Th2 polarization promotes B cell activation and increased antibody production, constituting a compensatory humoral response to the poor control of infection mediated by the deficient cellular response (105). However, sustained activation of the B-cell compartment can also lead to autoantibody generation, even in the absence of active infection, thereby increasing the risk of autoimmunity and tissue damage (105, 124). Studies in autoimmune diseases and chronic viral infections such as EBV and HIV have documented how loss of Th1 control can induce humoral dysregulation with pathological consequences (125, 126).

Consequently, although this humoral response seeks to assist in infection control, excessive Th2 and B cell activation is not only ineffective against intracellular pathogens but may also induce immune responses directed against self-tissues, exacerbating immune dysfunction (101).

#### 8.1.4 Increased aberrant HLA class II expression and autoimmunity

As inflammation persists, the high levels of IFN-γ produced associated with these ancestral haplotypes and the chronic inflammatory response induce aberrant HLA class II expression on nonprofessional antigen-presenting cells in peripheral tissues (i.e., in the cells of any tissue affected by inflammation), compared with less-reactive haplotypes (127–129). This process may increase the likelihood of presentation of autoantigens and neoantigens and the subsequent activation of autoreactive CD4+ T lymphocytes, which in turn initiate immune responses against host tissues (129, 130). At this point, CD4+ T cells, previously limited in their contribution to the immune response, begin to play a central role in perpetuating the inflammatory cycle (130). Autoreactive CD4+ T cells are activated and produce additional IFN-γ, further reinforcing inflammation and establishing a vicious cycle of tissue damage (130, 131).

Moreover, autoreactive B cells gain prominence because, by presenting autoantigens to autoreactive CD4+ T cells, they promote T cell activation and expansion, leading to pathogenic T–B cooperation (132). Once activated, CD4+ T cells provide co-stimulatory signals (such as CD40L) and cytokines that drive differentiation of B cells into autoantibody-producing plasma cells, amplifying the humoral response and chronic inflammation (133). The inflammatory cycle involves not only T and B cells but is also amplified by continuous production of proinflammatory cytokines (134). This process further intensifies chronic inflammation and contributes to disease progression in autoimmunity (135). Thus, activation of autoreactive CD4+ T and B lymphocytes generates an environment conducive to the emergence of autoimmune diseases.

Ancestral HLA-II haplotypes are associated with amplified pro-inflammatory cytokine responses, particularly IFN-γ, which can sustain a chronic inflammatory milieu and increase the risk of autoimmunity (1, 19, 20, 52). An example of this phenomenon was observed in a study using transgenic murine models expressing human HLA alleles (32). Specifically, two strains expressing HLA-DRB1*04:01 and HLA-DRB1*04:02, respectively, were compared, and although both developed immunity to influenza infection, only the *0401 mice generated a more intense cross-protective immunity (32). This response was associated with more pronounced activation of CD4+ T cells, including memory autoreactive Th17 cells against self-epitopes, suggesting an increased propensity for autoreactivity under conditions of intense immune activation (32).

#### 8.1.5 Immune exhaustion

As hyperactivity of CD8+ T cells persists, the phenomenon of immune exhaustion begins to develop (104). In this process, CD8+ T cells exposed to prolonged activation and stress undergo loss of function, limiting their ability to control infection effectively and perpetuating chronic inflammation (100, 101, 104). This exhaustion is characterized by expression of inhibitory markers such as programmed cell death protein 1 (PD-1), cytotoxic T-lymphocyte–associated protein 4 (CTLA-4), lymphocyte activation gene-3 (LAG-3) and T cell immunoglobulin and mucin domain-containing protein 3 (TIM-3), indicators of a functionally compromised state of CD8+ T cells (76, 104). In addition, NK cells responding to chronic inflammation may indirectly promote CD8^-^ T-cell exhaustion, thereby fostering viral persistence and immunological dysfunction (122).

The PD-1 receptor, in particular, is associated with inhibition of T cell activation, reducing their capacity to produce IFN-γ and other inflammatory cytokines (97, 104). This self-regulatory mechanism, although essential to prevent damage from excessive inflammation, also contributes to failure to eliminate pathogens that are latent or possess advanced immune-evasion mechanisms (97, 104, 123). With immune exhaustion, CD8^-^ T cells are unable to sustain effective responses, favoring infection persistence; the resulting prolonged antigen exposure may, in turn, increase susceptibility to autoimmunity (65, 76, 104, 136, 137).

### 8.2 Immunotherapies

Immune checkpoint inhibitors (ICIs), such as monoclonal antibodies directed against PD-1, programmed death-ligand 1 (PD-L1) and CTLA-4, have revolutionized cancer treatment by enhancing T cell activity against tumor cells (138). However, in individuals with ancestral HLA haplotypes (DR2-DQ6, DR4-DQ8 and DR3-DQ2), this therapeutic strategy can exacerbate underlying inflammatory mechanisms and increase the risk of autoimmunity, an adverse effect known as ICI-mediated immune toxicity (138–142).

In carriers of HLA haplotypes such as DR2-DQ6, DR4-DQ8 and DR3-DQ2, which evolved to mount robust proinflammatory responses to acute infections (1), blockade of PD-1 and CTLA-4 interferes with physiological immune-regulatory mechanisms that limit excessive inflammation (138–143).

By releasing the immunological “brake” represented by these molecules, ICIs induce hyperactivation of CD8+ and CD4+ T lymphocytes, resulting in an exacerbated cellular response with increased production of inflammatory cytokines such as IFN-γ, similar to the hyperresponse observed in carriers of ancestral haplotypes during acute infections, in comparison with other haplotypes (1, 144–146).

This increase in IFN-γ promotes aberrant HLA class II expression on nonprofessional antigen-presenting cells in peripheral tissues, facilitating presentation of autoantigens and activation of autoreactive CD4+ T lymphocytes (128, 129, 147). This process constitutes a critical point in the development of T cell–mediated autoimmunity (129). While ICIs can indeed reactivate pre-existing autoreactive lymphocytes, the higher incidence and severity of ICI-related autoimmunity in carriers of certain ancestral HLA-II haplotypes suggests that HLA genotype also modulates the generation, persistence, and effector phenotype of such clones, thereby increasing the likelihood that immune disinhibition induced by ICIs will precipitate clinically overt autoimmunity (138–142, 148–150). Recent clinical and translational studies indicate that specific HLA genotypes modulate the risk and phenotype of immune-related adverse events during checkpoint inhibitor therapy, suggesting a potential role for HLA profiling in toxicity risk stratification (151). Therefore, in individuals with these ancestral haplotypes, this inflammatory milieu can amplify chronic inflammation and lead to autoimmune diseases, partially recapitulating the mechanism observed in chronic infections with latent or highly immune-evasive pathogens (129, 138–143).

### 8.3 Vaccines

Vaccines are fundamental tools for prevention of infectious diseases (152). However, in individuals with ancestral HLA haplotypes such as DR2-DQ6, DR4-DQ8 and DR3-DQ2 they can trigger exacerbated inflammatory responses (20, 152–156). This phenomenon would occur especially in the context of vaccines that induce high levels of proinflammatory cytokine production, such as IFN-γ, generating an immunological environment that mimics the mechanisms that predispose to autoimmunity after infections, particularly in genetically predisposed individuals (1, 20, 152, 157).

Whereas DR2-DQ6 has solid clinical evidence, particularly in the case of post-Pandemrix^®^ narcolepsy following the H1N1 campaign, where carriers of the HLA-DQB1*06:02 allele showed a markedly increased risk associated with exacerbated IFN-γ responses and autoimmune/inflammatory syndrome induced by adjuvants (ASIA)-type phenomena, direct human vaccination studies are still lacking for DR3-DQ2 and DR4-DQ8 (152, 154, 155). Moreover, although not prophylactic vaccines against pathogens, transgenic murine models have shown that immunization with proinsulin peptides in the context of DR3-DQ2 induces autoimmune diabetes, and immunization with type II collagen in DR4-DQ8 carriers induces experimental arthritis (158, 159). These findings demonstrate how an “external” antigen can trigger autoimmunity in the presence of these haplotypes, a mechanism that could be emulated after vaccination with pathogen antigens that present similar amino-acid sequences (molecular mimicry) in susceptible individuals (152).

Carriers of ancestral HLA-II haplotypes tend to mount strong antigen-specific cellular responses, with marked IFN-γ production (1, 20). This evolutionary profile, optimized for combating acute infections, can amplify the risk of chronic inflammation and activation of autoreactive T lymphocytes in situations where a prolonged or exacerbated immune stimulus is generated, as might occur with certain vaccines (1, 20, 156). In these individuals, the interaction between genetics and vaccine design could predispose to development of T cell–mediated autoimmune diseases exacerbated by systemic inflammation (152, 156, 160).

mRNA-based and viral-vector vaccines differ significantly in design and the immunological impact they generate (161). mRNA vaccines, such as those developed for SARS-CoV-2, deliver a fragment of messenger RNA encapsulated in lipid nanoparticles that encodes a viral antigen (161). This type of vaccine has an approximate half-life of around 30 days in the organism, since the mRNA is degraded, limiting the duration of antigen expression (162). Although these vaccines elicit robust CD8+ T cell responses and substantial IFN-γ production, the transient nature of the stimulus minimizes the risk of sustained chronic inflammation (163). This is particularly relevant for individuals with ancestral HLA haplotypes, since the limited duration of the stimulus could reduce the probability of developing a persistent autoimmune response. Nevertheless, isolated cases of autoimmunity following mRNA vaccination have been reported, indicating that despite their more transient nature they are not entirely free of this risk (164, 165).

An example of this interaction was observed in individuals vaccinated against SARS-CoV-2: recent studies have shown that specific alleles such as HLA-DRB1*15:01 are associated with an increased T cell response to the spike antigen after the first and second vaccine doses, demonstrating a more intense immune response in carriers of this allele (166, 167). However, this association with enhanced immunogenicity does not establish a causal link to autoimmunity, which would require dedicated studies assessing post-vaccination autoimmune outcomes in carriers of these alleles.

On the other hand, viral-vector vaccines use attenuated or nonreplicating viruses, such as adenoviruses, to deliver genes encoding the viral antigen (168). Unlike mRNA vaccines, viral vectors can persist in the organism for a longer period, resulting in sustained immune stimulation (169, 170). This persistence may support antigen expression for months, so subsequent exposures can trigger potent T cell responses directed against antigen-expressing cells, which may translate into chronic tissue damage (171). Consequently, this prolonged stimulus could amplify IFN-γ production in individuals with ancestral HLA haplotypes, creating a chronic inflammatory environment that favors activation of autoreactive T lymphocytes and aberrant HLA-II expression in peripheral tissues (157, 167, 172–174). The latter phenomenon facilitates autoantigen presentation and perpetuates the inflammatory cycle, thereby increasing the risk of developing autoimmune diseases (157, 164, 173, 174).

It is important to clarify that not all vaccines with potent immunostimulatory capacity belong to the viral-vector category (175). A relevant example is Pandemrix^®^, the vaccine used during the H (176). This formulation can also generate a hyperimmune response, amplifying IFN-γ production in carriers of ancestral HLA haplotypes, which has been linked to autoimmune adverse events in genetically susceptible populations (154, 177, 178).

This concern materialized in a paradigmatic case during mass vaccination with Pandemrix^®^ against H1N1 (177). In Finland and Sweden, a marked increase in narcolepsy cases was reported among adolescents and young adults, particularly in carriers of the HLA-DQB1*06:02 allele (154, 177). Presence of this allele was strongly associated with increased risk of narcolepsy in vaccine-exposed cohorts, even in heterozygotes, consistent with an interaction between genetic profile and vaccine-associated risk (154). Studies also indicated that anti-A/H1N1 antibody levels were significantly higher in individuals younger than 13 years, and that both patients and HLA-DQB1*06:02-positive controls showed an increased serological response (154, 177). Epidemiologic evidence for vaccine-associated autoimmunity is strongest in specific, well-characterized episodes (e.g., Pandemrix-associated narcolepsy), whereas most other vaccine–autoimmunity links remain limited to case reports or small series. Hence, generalization should be cautious: large, HLA-stratified vaccine safety cohorts are necessary to quantify absolute and relative risks across haplotypes.

In addition to intrinsic vaccine design, adjuvants included in many vaccine formulations constitute a critical factor, especially in predisposed individuals (157, 179). These compounds act via the “adjuvant effect”: coadministration of an antigen with microbial-derived components activates Toll-like receptors (TLRs) on antigen-presenting cells (APCs), which not only induces release of proinflammatory cytokines but also increases expression of antigen-presentation molecules such as HLA-II and of co-stimulatory molecules such as B7-1/2 (CD80/CD86), even on nonprofessional cells (for example, fibroblasts or endothelial cells) (157, 180). In the case of classical adjuvants such as aluminum salts, designed to produce localized inflammation at the injection site, this activation enhances IFN-γ and other proinflammatory cytokine production (181, 182). In carriers of ancestral HLA haplotypes (DR2-DQ6, DR3-DQ2, DR4-DQ8), whose baseline inflammatory response is already more intense, such increases in HLA-II and costimulatory signaling could trigger aberrant IFN-γ production and expansion of autoreactive T cells, emulating an autoimmune process similar to that induced by chronic infections (20, 65, 153, 183, 184). Likewise, more advanced adjuvants, such as oil-in-water emulsions like MF59 or TLR4 agonists, may further exacerbate this disproportionate immune activation, increasing the risk of autoimmunity in these individuals (157, 180, 185, 186).

The prolonged stimulus generated by viral-vector vaccines and certain adjuvants can reproduce features of chronic infection in terms of sustained inflammation (65, 157, 171, 187). In individuals with ancestral HLA haplotypes, this prolonged inflammation could induce aberrant HLA-II expression in nonimmune cells and activate autoreactive T cells, creating an inflammatory milieu that perpetuates tissue damage (20, 153, 157). This process contributes to the development of autoimmune conditions such as inflammatory arthritis, myocarditis, autoimmune encephalitis and autoimmune hepatitis (157, 179, 188, 189).

Although vaccines are essential for control of infectious diseases, they can trigger dysregulated immune responses in individuals with ancestral HLA haplotypes because of these individuals’ tendency to generate intense inflammation. Therefore, vaccine design, adjuvant selection and post-vaccination monitoring of genetically susceptible subjects are crucial to minimize the risk of vaccine-induced autoimmunity. While mRNA vaccines may appear safer due to their transient antigenic stimulus, clinical cases of autoimmunity following their use demonstrate that genetic predisposition is the primary factor, with antigen-exposure duration acting as a secondary modulator of an immune process preconditioned by the host genome.

### 8.4 Allergies

Chronic exposure to allergens can induce a persistent inflammatory milieu, especially in genetically predisposed individuals, which may favor development of autoimmune diseases (20, 190, 191). Sensitization to allergens responsible for allergic rhinitis, asthma or dermatitis involves an exaggerated immune response mediated by CD4+ T cells and B cells (192, 193). This activation generates a Th2 response characterized by production of cytokines such as IL-4, IL-5 and IL-13, promoting chronic inflammation in the airways, skin or other affected tissues (192, 194). However, in individuals with ancestral HLA-II haplotypes such as DR2-DQ6, DR4-DQ8 or DR3-DQ2, the immune response can diverge toward a more intense Th1 profile, increasing production of IFN-γ and TNF-α (20, 153, 191). Immunogenetic studies have shown that alleles such as HLA-DRB1*15, HLA-DRB1*03 and HLA-DRB1*04 are significantly associated with allergen-specific IgE responses to diverse allergens, supporting their direct involvement in allergic processes (195–197). This suggests that, in carriers of these haplotypes, chronic allergen exposure could induce more intense and sustained inflammation that may create a milieu favorable to autoimmunity (20, 191).

The study by Germundson et al. (2022) supports the influence of HLA-II alleles on allergic responses, showing differences in antibody production and inflammation according to the expressed allele (198). It was observed that both male and female HLA-DRB1*03:01 mice, as well as female HLA-DRB1*15:01 mice, exhibited significantly increased β-lactoglobulin (BLG)-specific IgE production, indicating a more intense allergic response (198). Conversely, sensitized HLA-DQ8 mice (HLA-DQA1*03:01-DQB1*03:02) of both sexes, and to a lesser extent male HLA-DRB1*15:01 mice, showed robust increases in BLG-specific IgG1, suggesting a different but equally relevant immune response in allergic sensitization (198). Additionally, sensitized HLA-DRB1*15:01 mice displayed sex-specific behavioral changes, with males showing altered mobility and anxiety-like behavior, while females exhibited impaired spatial memory. A significant shortening of villi was also detected in sensitized males carrying HLA-DRB1*03:01 or HLA-DRB1*15:01 alleles (198). Even the HLA-DRB1*15:01-DQB1*06:02 haplotype was associated with cow’s-milk allergy, particularly with higher humoral responses to BLG (199). This suggests that, in carriers of these haplotypes, chronic allergen exposure could induce more intense and sustained inflammation that may promote an environment favorable to autoimmunity (20, 191, 198).

Persistent inflammation can erode immunological tolerance, promoting the activation of autoreactive T cells and the production of autoantibodies by B cells (200, 201). Proinflammatory mediators, notably IFN-γ, can upregulate HLA-II on non-professional antigen-presenting cells, thereby facilitating local presentation of self-antigens, including neoantigens, and focal immune activation (200, 202, 203). In combination with tissue dysfunction and co-factors (e.g., chronic infections, metabolic stress, or physical trauma), this milieu favors loss of tolerance and the emergence of autoimmune pathology (201, 204–206).

### 8.5 Obesity

Obesity, as a state of low-grade chronic inflammation, can mimic the inflammatory responses observed in persistent infections and increase expression of HLA class II on nonprofessional antigen-presenting cells (207–211).

Hyperlipidemia, a feature of obesity, activates Toll-like receptors (TLRs), particularly TLR4, in macrophages and dendritic cells, stimulating NF-κB signaling and the production of cytokines such as TNF-α, IL-6 and IFN-γ (208). Similarly, hyperglycemia induces oxidative stress and endoplasmic reticulum stress, which activate the unfolded protein response (UPR) and perpetuate inflammatory signaling (208). The combination of these pathways amplifies systemic inflammation, which in individuals carrying ancestral HLA class II haplotypes could enhance HLA-II expression in peripheral tissues and facilitate activation of autoreactive T lymphocytes (153).

Moreover, increased intestinal permeability in obesity permits translocation of lipopolysaccharide (LPS) into the systemic circulation, triggering metabolic endotoxemia (208). LPS activate TLR4, exacerbating NF-κB–mediated inflammation (208). In carriers of ancestral haplotypes, this persistent inflammation could drive dysregulated activation of T and B cells, perpetuating an inflammatory cycle that favors development of autoimmune diseases (153).

The chronic inflammatory state derived from obesity sustains IFN-γ production (211), which in proinflammatory ancestral haplotypes prone to intense immune responses and may further amplify HLA class II expression on non-immune cells (153). This phenomenon, documented in HLA-transgenic models and genetic studies, could facilitate autoantigen presentation and promote activation of autoreactive T cells (17). This inflammatory cycle is particularly detrimental in genetically predisposed individuals, where metabolic stress and systemic inflammation potentiate progression toward autoimmune disease (17, 153).

Therefore, in the context of obesity, individuals with proinflammatory HLA class II haplotypes may experience amplification of systemic inflammation, aberrant HLA-II expression and an increased likelihood of developing autoimmune diseases (153, 207, 211). Moreover, epidemiological data show that excess adiposity can synergize with HLA risk alleles to raise autoimmune risk, most notably adolescent obesity interacting with HLA-DRB1*15 to increase multiple sclerosis susceptibility, and overweight/obesity interacting with high-risk HLA genotypes to promote latent autoimmune diabetes in adults (212, 213).

### 8.6 Chronic physical stress

Chronic physical stress, such as recurrent physical trauma, hypoxia or persistent injury, generates a sustained inflammatory state that activates NF-κB signaling (214, 215). This activation, mediated by proinflammatory cytokines such as TNF-α and IL-1, leads to increased production of IFN-γ, primarily mediated by T lymphocytes and natural killer (NK) cells (216, 217). IFN-γ in turn activates the JAK–STAT pathway, especially STAT1, which regulates transcription of inflammatory genes and HLA-II expression, thereby amplifying the immune response (218, 219). NF-κB and STAT1 signaling pathways thus act in concert to potentiate chronic inflammation (220, 221). In carriers of ancestral HLA class II haplotypes such as DR2-DQ6, DR4-DQ8 and DR3-DQ2, this overproduction of IFN-γ and concurrent activation of both pathways could intensify inflammatory responses due to greater genetic sensitivity to these mediators (1, 20, 52).

Although physical stress and tissue trauma have been linked to the onset or exacerbation of autoimmune diseases (e.g., psoriasis, psoriatic arthritis, rheumatoid arthritis) (205, 222), direct evidence of interaction between specific HLA-II haplotypes and physical stress is limited. Notably, a large Swedish study reported a synergistic effect between head trauma and HLA-DRB1*15:01 on multiple sclerosis risk, supporting the concept that HLA genotype can modulate the autoimmune consequences of tissue injury (223).

### 8.7 Transplantation

In the transplantation setting, the presence of proinflammatory HLA class II haplotypes, such as DR4-DQ8, DR2-DQ6 and DR3-DQ2, has been associated with increased production of proinflammatory cytokines after the procedure, promoting a persistent systemic inflammatory milieu (19, 224). This exacerbated immunological profile can intensify activation of T and B cells, increase the risk of acute and chronic graft rejection, and contribute to post-transplant tissue injury (224–226).

Several studies have reported that carriers of these haplotypes exhibit higher levels of mediators such as IFN-γ, TNF-α and IL-6, together with sustained activation of NF-κB and STAT1 pathways, thereby potentiating the inflammatory response (19, 20, 52, 153, 227). This hyperreactive immune state not only compromises graft function but has also been associated with higher post-transplant morbidity and mortality, underscoring the prognostic importance of HLA genetic profile in clinical outcomes (19, 224). For example, pancreas transplants in patients carrying HLA-DR3 and/or HLA-DR4 have shown higher risk of recurrence of type 1 diabetes (226).

## 9 Discussion

The relationship between HLA haplotypes and the immune response represents a central framework for understanding how evolution has shaped both defense against lethal infections and current susceptibility to inflammatory and autoimmune diseases (1, 20, 22). Ancestral HLA class II haplotypes (DR2-DQ6, DR4-DQ8 and DR3-DQ2) have been selected for their ability to induce rapid and intense immune responses to acute infections, mediated primarily by production of proinflammatory Th1 cytokines such as IFN-γ and TNF-α (1, 19, 20, 52). This adaptive advantage, which in historical contexts may have meant the difference between survival and death during high-mortality epidemics, can today translate into an increased propensity for exaggerated immune responses, systemic inflammation and autoimmunity (1, 2, 17–20, 22, 228, 229). The conceptual model depicted in **Figure 1** integrates these evolutionary pressures with the mechanistic pathways that may link ancestral HLA-II haplotypes to immune hyperreactivity and autoimmunity.

This hyperreactivity may be related to the high promiscuity of antigen presentation by the DR2-DQ6, DR4-DQ8 and DR3-DQ2 haplotypes, which permits recognition of a broad repertoire of both pathogenic and self-peptides; this enhances their efficacy in clearing acute infections but raises the risk of autoimmunity in contexts of persistent antigenic exposure, such as chronic infections (1, 20).

In the modern environment, characterized by lower mortality from acute infections but high persistence of pathogens with sophisticated immune-evasion strategies, such as *Mycobacterium tuberculosis*, Epstein–Barr virus or citomegalovirus, immunological hyperreactivity can become a double-edged sword (81–86, 92, 95). The intense inflammatory response, although initially protective, can be maintained chronically without achieving pathogen eradication, generating a persistent microenvironment that promotes loss of immune tolerance and autoimmunity (1, 19, 20). Mechanisms such as molecular mimicry, bystander activation and antigenic persistence can convert an initially protective response into a pathogenic pathway that promotes autoimmunity in genetically susceptible individuals (230). Sustained T cell activation and continuous production of proinflammatory cytokines, such as IFN-γ, sustain a systemic inflammatory state that causes tissue damage (1). In this context, the immune response not only fails to eliminate the infection but may also contribute to pathogen persistence and to development of autoimmune diseases, as observed in multiple sclerosis, active tuberculosis or visceral leishmaniasis (1, 20, 37, 53, 85).

This same mechanism could extend beyond classical autoimmune diseases (such as multiple sclerosis, rheumatoid arthritis, lupus, celiac disease or type 1 diabetes) and might explain increased susceptibility to conditions characterized by systemic inflammation and immune dysregulation (**Table 1**), such as long COVID, myalgic encephalomyelitis/chronic fatigue syndrome and certain post-vaccination syndromes (179, 231–239). In all of these conditions, immunological profiles have been described that include sustained activation of T and B lymphocytes, increased IFN-γ, autoantibody production and ectopic, sustained HLA-II expression (which could be more pronounced compared with less-reactive haplotypes), mechanisms that are congruent with the pathophysiology observed in carriers of these haplotypes (234, 235, 238, 240–247). Several studies have even linked the development of post-vaccination syndromes and ME/CFS with alleles belonging to the DR2-DQ6, DR4-DQ8 and DR3-DQ2 haplotypes (184, 239, 248–252). In the case of long COVID, for example, although some genetic analyses suggest involvement of the HLA class II region, robust and replicated studies conclusively linking the DR2-DQ6, DR4-DQ8 or DR3-DQ2 haplotypes to long COVID risk are still lacking (21, 253). However, haplotypes such as HLA-DRB1*15:01-DQA1*01:02-DQB1*06:02 (DR2-DQ6) have been shown to bind numerous peptides from the SARS-CoV-2 spike protein with high affinity, which favors stronger immune responses (166, 254–257). This hyperreactivity, although beneficial against extracellular pathogens, has also been associated with higher autoimmune risk (153), suggesting a possible indirect role of these haplotypes in susceptibility to long COVID. Indeed, HLA-DRB1*15:01 has also been associated with increased risk of severe COVID-19 (258).

**Table 1:** Autoimmune and inflammatory diseases associated with ancestral HLA-II haplotypes.

Haplotypes	Main risk associations	Refs	Main protective associations	Refs
DR2-DQ6 (DRB1*1501, DQA1*0102, DQB1*0602)	Systemic lupus erythematosus	(322–325)	Type 1 diabetes mellitus	(326–329)
	Sjögren’s syndrome	(330, 331)		
	Multiple sclerosis	(332, 333)		
	Myalgic encephalomyelitis/Chronic fatigue syndrome	(334, 335)		
	Late-onset/acquired myasthenia gravis	(336, 337)		
	Fulminant type 1 diabetes	(338)		
	Sarcoidosis	(339, 340)		
	Post-transplant complications	(19)		
	Narcolepsy	(341)		
	Narcolepsy post-Pandemrix^®^	(155, 246)		
	Chronic Lyme arthritis	(293)		
	Autoimmune/Inflammatory Syndrome Induced by Adjuvants (ASIA)	(155, 156)		
	Autoimmune adverse events secondary to immunotherapy (e.g., irAEs from ICIs)	(139–142)		
	Autoimmunity against the muscarinic M3 acetylcholine receptor	(283)		
DR3-DQ2 (DRB1*0301, DQA1*0501, DQB1*0201)	Systemic lupus erythematosus	(342)	No consistent protective association reported	—
	Multiple sclerosis	(17)		
	Type 1 diabetes mellitus	(343, 344)		
	Celiac disease	(345–347)		
	Graves’ disease	(348, 349)		
	Sjögren’s syndrome	(350, 351)		
	Early-onset myasthenia gravis	(352–354)		
	Addison’s disease	(355, 356)		
	Type I autoimmune hepatitis	(357)		
	Neuromyelitis optica	(358)		
	Post-transplant complications	(19)		
	Autoimmune adverse events secondary to immunotherapy (e.g., irAEs from ICIs)	(138, 140)		
DR4-DQ8 (DRB1*04, DQA1*03, DQB1*0302)	Multiple sclerosis	(359)	No consistent protective association reported	—
	Type 1 diabetes mellitus	(360, 361)		
	Celiac disease	(362–364)		
	Rheumatoid arthritis	(130, 365)		
	Hashimoto’s thyroiditis	(366, 367)		
	Addison’s disease	(355, 356)		
	Type I autoimmune hepatitis	(357)		
	ANCA-associated vasculitis	(368)		
	Chronic Lyme arthritis	(293)		
	Post-transplant complications	(19)		
	Autoimmune adverse events secondary to immunotherapy (e.g., irAEs from ICIs)	(138, 140, 141)		
Other protective HLA alleles	—	—	DRB1*13 (protection across several autoimmune diseases)	(369–371)
			DERAA-containing alleles (protection in ACPA-positive rheumatoid arthritis in some studies)	(372, 373)

Summary of autoimmune and hyperinflammatory conditions linked to the ancestral haplotypes DR2–DQ6, DR3–DQ2, and DR4–DQ8, highlighting their role as genetic predisposition factors for immune hyperreactivity and loss of tolerance. Protective associations are markedly fewer than risk associations; the most consistently replicated protective effect is DQB1*06:02 (part of DR2–DQ6) against classic, autoantibody-positive type 1 diabetes. Other protective alleles, including HLA-DRB1*13 (notably 13:01 and 13:02) and DERAA-encoding DRB1 alleles (e.g., DRB101:03, *04:02, *11:02, *11:03, *13:01, 13:02), are associated with reduced susceptibility and/or milder disease phenotypes (e.g., less erosive rheumatoid arthritis). These protective effects are disease- and population-specific, and exceptions have been reported in certain contexts.

Beyond these associations, the pathophysiology of post-viral syndromes such as long COVID and ME/CFS may require integration of neuroendocrine and autonomic mechanisms to explain the constellation of profound fatigue, cognitive dysfunction, exertional intolerance and dysautonomia present in many patients (259–263). In this regard, persistent antigenic stimulation in genetically susceptible hosts (HLA-II) could favor the development of autoimmunity directed against endocrine and autonomic targets (21, 230, 248, 253, 264, 265); this is especially relevant for understanding the clinical presentation of long COVID and ME/CFS (261, 266, 267). Frequently, and not mutually exclusively but rather complementarily, two pathogenic pathways coexist and converge on the same clinical phenotype:

- Autoimmunity directed at the hypothalamic–pituitary–adrenal (HPA) axis and hypocortisolism (261). Cortisol, produced by the adrenal glands under control of the HPA axis, is the principal endogenous anti-inflammatory regulator (268). The HPA axis may be affected by direct viral injury, by neuroinflammation, or by autoimmunity against the pituitary (hypophysitis) following infection, leading to central or functional hypocortisolism (269–274). Relative and sustained hypocortisolism diminishes the capacity to suppress inflammatory responses, promotes neuroinflammation, and reduces tolerance to exertion and stress, thereby perpetuating the chronic inflammation observed in long COVID and ME/CFS (260–262).
- Autoimmunity against cholinergic receptors and loss of parasympathetic tone (266, 267, 275–277). Acetylcholine released by the vagus nerve constitutes a potent anti-inflammatory pathway (278, 279); the emergence of autoantibodies or antigen-specific T-cell responses directed against muscarinic (e.g., M3) or nicotinic receptors can block cholinergic signaling and reduce the “vagal brake” (280–284). This leads to dysautonomia (POTS, orthostatic intolerance), relatively increased sympathetic activity and loss of inflammatory control, conditions that facilitate chronification of the immune response (267, 277, 285). Recent data show associations between M3-reactive responses and certain HLA-DR genotypes, suggesting possible relevance of haplotypes such as HLA-DRB1*15:01–DQA1*01:02–DQB1*06:02 (DR2-DQ6) in this context (283). Moreover, the clinical benefit observed in some studies with cholinergic modulators (e.g., pyridostigmine) supports the pathophysiological plausibility of this pathway (286–289).

These mechanisms are not mutually exclusive and frequently potentiate one another (279). Loss of vagal tone favors sympathetic predominance which, in settings of sustained activation, can ultimately uncouple or attenuate the response of the HPA axis (279, 290, 291). Conversely, a sustained state of hypocortisolism reduces hormonal control of inflammation and may diminish cholinergic modulation, closing a pro-inflammatory feedback loop that facilitates chronification and immune exhaustion (261, 279, 292). Therefore, HLA-II susceptibility could facilitate viral persistence or reactivation (e.g., EBV, SARS-CoV-2, other herpesviruses), fueling continuous antigenic stimulation or neoantigen exposure that promotes generation of autoantibodies or autoreactive T cell responses directed against the pituitary, cholinergic receptors or other neuronal targets, thereby closing the pathogenic circuit. The same pattern can be observed in other clinical models: in chronic Lyme arthritis (*Borrelia burgdorferi*), HLA-DR4 alleles (and to a lesser extent DR2) are overrepresented in chronic, refractory forms, suggesting that these lineages favor both immune-mediated persistence of infection and development of joint autoimmunity (293, 294). Similarly, in multiple sclerosis the risk haplotype HLA-DRB1*15:01 (DR2) is associated with impaired immune control of EBV, hindering recognition and elimination of EBV-infected cells (53) and, together with EBV immune-evasion factors (vIL-10, BNLF2a) that reduce antigen presentation (62), promotes viral persistence and chronic antigenic stimulation that can trigger reactivity against myelin (295, 296). Taken together, these models illustrate how an HLA-II predisposition that facilitates microbial persistence can, through persistent antigenic or neoantigen presentation, secondarily trigger autoimmunity (arthritis in Lyme disease; demyelination in multiple sclerosis) and suggest that similar mechanisms may be operating in long COVID, myalgic encephalomyelitis/CFS and certain post-vaccination syndromes. However, although mechanistic models (viral immune evasion plus hyperinflammatory host responses) are plausible and supported by animal and *in vitro* work, human data remain largely associative; prospective studies linking allele-specific antigen presentation, pathogen load/persistence and downstream autoimmunity are still needed to move from correlation to causation.

Far from representing an inherent “weakness” of ancestral haplotypes, this phenomenon suggests an evolutionary trade-off in which the same immune response that conferred an advantage against lethal infections under high infectious-mortality conditions may be counterproductive in the modern environment (1, 20, 22, 228). Rather than an intrinsic deficiency in defense against chronic infections, what occurs is a mismatch between pathogen characteristics and the immune response induced by these haplotypes (61, 228, 229, 297). Intracellular pathogens that possess advanced immune-evasion mechanisms require more nuanced, less hyperreactive control strategies, which may account for the relative inefficacy of highly reactive HLA haplotypes in chronic infection contexts (1, 20, 123, 298).

It is important to note that the persistence of these haplotypes in the human population reflects an adaptive equilibrium (1, 20). While DR2-DQ6, DR4-DQ8 and DR3-DQ2 confer an advantage against acute infections by generating more effective immune responses, this same mechanism renders carriers more prone to exaggerated immune responses in chronic infections, increasing autoimmune disease risk (**Table 2**) (1, 20, 22, 61, 228). This balance between protection from acute infections and risk of autoimmunity following chronic infection may have been crucial for survival in earlier environments where lethal infections were far more common than chronic infections (61, 299–302). These observations provide indirect support for an evolutionary trade-off between infection resistance and autoimmune susceptibility; however, genomic signals can be influenced by demographic factors and association-study biases (e.g., overrepresentation of European populations), so functional validation is required for each allele and context.

**Table 2:** Summary schematic of major HLA class II haplotype associations with autoimmune diseases.

Haplotype	MS	T1D	Celiac disease	RA	Narcolepsy	SLE	Sjögren’s syndrome	Graves’ disease	Hashimoto’s thyroiditis	Addison’s disease	Myasthenia gravis
DR2–DQ6	↑ strong	↓ strong protection	—	—	↑ strong	± moderate	↑ moderate	± reported	—	± (post-transplant/limited)	↑ moderate (late-onset MG)
DR3–DQ2	± (population-dependent)	↑ strong	↑ strong	—	—	↑ moderate	↑ moderate–strong	↑ strong	↑ strong (shared with DR4)	↑ strong	↑ strong (early-onset MG)
DR4–DQ8	± (allele-dependent)	↑ strong	± (less than DR3)	↑ strong (shared-epitope alleles)	—	± reported	± less consistent	↑ (shared with DR3)	↑ strong (overlap with DR3)	↑ strong	± (subtype-specific)

↑ = consistent/replicated association  ± = reported but inconsistent (population- or allele-dependent)

↓ = consistent protective association

Summary schematic of the main autoimmune diseases most frequently associated with ancestral HLA class II haplotypes (DR2–DQ6, DR3–DQ2, and DR4–DQ8). The table highlights relative strength and direction of genetic association across major autoimmune conditions. Protective effects are comparatively rare, with DQB1*06:02 (within DR2–DQ6) showing the most reproducible protection against classic, autoantibody-positive type 1 diabetes. Associations may vary depending on allele subtypes, ethnicity, and disease phenotype (see **Table 1** for detailed references). MS, multiple sclerosis; T1D, type 1 diabetes; CD, celiac disease; RA, rheumatoid arthritis; SLE, systemic lupus erythematosus; SS, Sjögren’s syndrome; GD, Graves’ disease; HT, Hashimoto’s thyroiditis; AD, Addison’s disease; MG, myasthenia gravis.

Therefore, the evolution of these HLA haplotypes must be understood in terms of historical evolutionary fitness. The advantage conferred by rapid clearance of acute infections likely outweighed the costs associated with autoimmune diseases, which were less prevalent in populations historically exposed to high infectious mortality (1). In this sense, the persistence of these haplotypes reflects an adaptation that favored short-term survival against immediate threats posed by virulent, lethal pathogens, despite adverse long-term health consequences.

**Figure 3:**
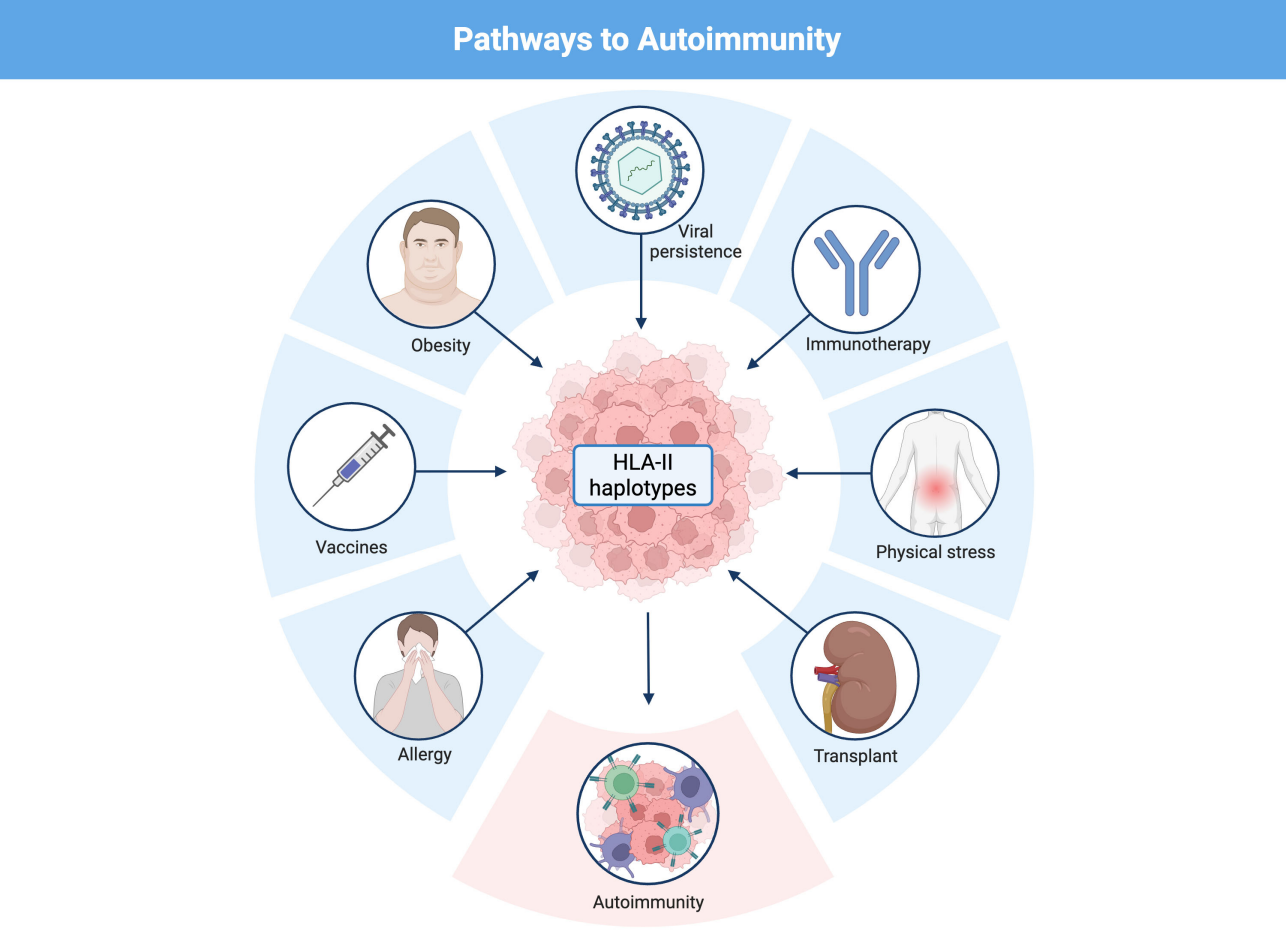
Pathways linking ancestral HLA-II haplotypes to autoimmunity. Schematic representation of the interplay between genetic predisposition and chronic immune activation in the development of autoimmunity. Ancestral HLA-II haplotypes (DR2-DQ6, DR3-DQ2, DR4-DQ8) confer evolutionary advantages against acute infections by promoting strong T cell responses, but in the modern context they predispose to immune hyperreactivity. Persistent antigenic stimulation, often derived from intracellular pathogens such as viral infections (e.g., Epstein–Barr virus), but also triggered by vaccines, immunotherapy, obesity, chronic physical stress, allergy, or transplantation, can sustain proinflammatory Th1/Th17 responses, continuous IFN-γ production, and aberrant HLA-II expression in peripheral tissues. In genetically susceptible individuals, this persistent inflammatory microenvironment promotes loss of tolerance, activation of autoreactive T and B cells, and the development of autoimmune disease.

Hence, the persistence of these haplotypes in modern populations, with particularly high frequencies in northern Europe and Scandinavia (23–25, 303), reflects this evolutionary balance where the benefit against acute infections outweighed the cost of autoimmunity, which often manifested after reproductive age (1, 61, 302, 304, 305). Today, however, shifts in pathogen ecology, increased life expectancy and exposure to novel chronic inflammatory stimuli, such as latent infections, vaccinations, obesity or chronic stress, have converted these adaptive advantages into pathophysiological vulnerabilities (**Figure 3**) (306–310).

This phenomenon highlights the complexity of immune system evolution, where the same immune response that is protective in an acute infection context can be detrimental in the setting of chronic infections (1, 61). Modern interventions in the form of immunomodulatory therapies and treatments for autoimmune diseases may help mitigate the negative effects of this immune hyperreactivity, but they also underscore the need for a more holistic approach that considers the evolutionary history of the human immune system (2, 311).

## 10 Concluding remarks and future directions

The paradoxical “weakness” of ancestral HLA haplotypes with respect to chronic infections should not be interpreted as an immunological deficiency but rather as an evolutionary trade-off whereby the same immunological trait that ensured survival against lethal pathogens in the past can now increase the risk of autoimmune disease in the present (1, 19, 20, 61). This balance between protection and risk reflects how evolution has shaped the human immune system to confront the infectious threats of its time, and raises questions about how changes in pathogen environments affect human health today.

Understanding this duality allows not only a reinterpretation of autoimmunity as the consequence of an evolutionary *trade-off* but also the proposal of more personalized preventive and therapeutic strategies. In the future, integrating HLA-II profiling into risk assessment could help identify individuals susceptible not only to classical autoimmune diseases but also to post-infectious or post-vaccinal inflammatory syndromes, allowing adjustment of immunological stimulus intensity and development of safer, more effective interventions.

Progress will require a multidimensional, prioritized approach that moves from association to mechanism and enables safe, HLA-informed translation into the clinic (26, 312). First, well-powered, multi-ancestry prospective cohorts with high-resolution HLA sequencing and harmonized phenotyping are essential to define population- and ancestry-specific effects and to reduce Eurocentric bias in current datasets (26). Second, allele-resolved, paired immunopeptidomics on affected and matched control tissues (or validated surrogate tissues) should be used to determine whether implicated haplotypes present broader or disease-relevant peptide repertoires *in vivo*; recent methodological advances make these experiments increasingly feasible on limited clinical samples (15, 313). Third, integrate improved in-silico HLA-II presentation models with empirical peptidomics to prioritize candidate peptides for downstream testing, contemporary predictors have substantially improved performance across HLA-DR/DQ/DP loci and can reduce the candidate search space for functional assays (314, 315). Fourth, deploy high-throughput antigen/TCR discovery platforms and pooled functional screens (e.g., TCR-mapping and barcoded peptide/TCR assays) to validate T-cell reactivity and establish whether peptides presented by specific alleles elicit pathogenic autoreactive T-cell responses (316, 317). Finally, complement molecular work with translational studies that include HLA stratification in vaccine surveillance and immunotherapy cohorts, paired single-cell/spatial transcriptomics and standardized longitudinal phenotyping; integrate data into open, interoperable resources so findings are reproducible and can inform HLA-guided preventive or therapeutic strategies (318–321).

This work has important limitations: much of the evidence is correlational (genomic signals and population associations) and may be affected by demographic factors, population structure and biases in association studies (e.g., overrepresentation of European populations). In addition, HLA allele imputation (statistical estimation of variants not directly genotyped) and heterogeneity in clinical definitions of post-infectious phenotypes (e.g., long COVID) limit comparability between studies; therefore, the evolutionary and mechanistic hypotheses discussed here require allele- and context-specific functional validation.
